# Fractal Dimension Analysis of Mandibular Trabecular Architecture in Gingival Recession During Orthodontic Retention: A Cross-Sectional Study

**DOI:** 10.3390/diagnostics15081013

**Published:** 2025-04-16

**Authors:** Merve Küçükoğlu Çolak, Resul Çolak, Orhan Cicek

**Affiliations:** 1 Department of Periodontics, Faculty of Dentistry, Zonguldak Bülent Ecevit University, Zonguldak 67600, Türkiye; 2 Department of Orthodontics, Faculty of Dentistry, Zonguldak Bülent Ecevit University, Zonguldak 67600, Türkiye; orhancicek@beun.edu.tr

**Keywords:** orthodontic treatment, retention, gingival recession, gingival index, plaque index, fractal dimension analysis

## Abstract

**Objectives**: This study used fractal dimension (FD) analysis to evaluate alveolar trabecular changes associated with gingival recessions in the mandibular incisor-canine and premolar regions in the post-orthodontic retention period, compare them to non-recession regions, and assess their correlation with plaque (PI) and gingival indices (GIs). **Methods**: This cross-sectional case–control study included 60 patients with mandibular gingival recession (35 females, 25 males; mean age: 21.91 ± 2.59 years), with apical trabecular bone regions of interest (ROIs) identified in the incisor-canine and premolar regions. Patients were divided into four groups based on the regions of recession, with non-recessional regions in the contralateral quadrant considered as control regions. FD analysis was performed on the specified ROIs using panoramic radiographs taken before treatment (T0), after treatment (T1), and in the retention period (T2). Patients’ PI and GI scores and incisor-mandibular plane angles (IMPAs) were recorded. **Results**: The FD values significantly decreased in Groups 1 and 2 (left and right incisor-canine regions) (*p* < 0.05), but no significant changes were found in Groups 3 and 4 (left and right premolar regions) and the control group. The FD values showed a significant correlation with the PI and GI scores (*p* < 0.05), but no correlation was found with IMPAs (*p* > 0.05). **Conclusions**: Alterations in alveolar trabeculation in gingival recession can be assessed by FD analysis. Decreased FD values correlate with worsening oral hygiene and higher PI and GI scores. Changes in the IMPA do not correlate with changes in FD, highlighting the importance of regular periodontal check-ups after orthodontic treatment.

## 1. Introduction

Gingival recession is defined as the migration of the gingival margin from the cemento-enamel junction toward the apical region, resulting in exposure of the root surface [1]. Epidemiologic studies have shown its widespread prevalence [2], particularly in the mandibular incisors [3]. The etiology is multifactorial and includes predisposing factors [4] such as a thin periodontal biotype, alveolar bone dehiscence, and malocclusion, as well as precipitating factors such as mechanical trauma from improper brushing, orthodontic tooth movement, and periodontal inflammation [5].

Malocclusion can compromise alveolar bone support and increase the risk of gingival recession, especially in vestibularly positioned teeth, while proper tooth alignment improves oral hygiene and reduces recession associated with periodontal inflammation [6]. Although orthodontic treatment can mitigate some risk factors associated with gingival recession, recent systematic reviews have also linked it to an increased incidence of recession [7,8,9,10]. Orthodontic forces may displace tooth roots closer to or beyond the alveolar cortical bone layer, potentially leading to alveolar bone dehiscence [11,12,13].

Fractal dimension (FD) analysis has been used in previous studies to quantitatively assess trabecular changes in alveolar bone [14,15,16]. This technique detects complex structural patterns in trabecular bone and quantitatively expresses the complexity of the bone with a measure called FD [17]. FD describes the roughness of the texture by capturing the self-similarity of the gray value variations within the texture [18,19]. A high FD value indicates dense and complex bone tissue with well-defined trabecular plates, while a low FD value signifies reduced mineralization with less density and complexity [20,21]. FD analysis is a non-invasive, reproducible method for assessing scale-invariant structures in radiographs and is unaffected by exposure differences, minor orientation variations, and region of interest (ROI) selection [22,23,24,25]. It is therefore particularly suitable for analyzing trabecular bone patterns and detecting changes in alveolar bone mineralization in clinical studies [18,26,27,28]. In addition, it has the potential to provide critical insights into the diagnosis, treatment planning, and prognosis of alveolar bone response in gingival recession cases [28].

Although orthodontic treatment improves dental alignment and periodontal conditions, optimal periodontal health requires professional oral hygiene motivation and effective plaque control [29,30]. However, whereas factors such as a thin periodontal phenotype, age, and smoking history have been reported to contribute to the gingival recession observed in the retention period [31], it has also been suggested that mandibular incisor protrusion and the type of retention appliance may play a role [7,32,33]. In addition, even though uncontrolled tooth movement caused by active or deformed fixed retainers can lead to undesirable gingival recession [34,35], studies have shown conflicting results as to whether fixed retention may be considered a risk factor for the development of gingival recession [31,36,37].

As a predisposing factor, alveolar bone dehiscence may develop due to orthodontic treatment, further contributing to gingival recession in affected areas [4,5]. Although bone dehiscence may cause a gingival recession, it may also cause changes in bone mass and a disruption in the microarchitecture [4,5]. Therefore, evaluating the reciprocal changes in the gingiva and bone is essential for obtaining a comprehensive understanding of gingival recession, which is influenced by multiple factors.

In the current literature, although the relationship between various factors and undesirable periodontal problems occurring during the orthodontic retention period has been investigated [32,38,39], no study has addressed the relationship between these problems and the fractal dimensions of the mandibular alveolar bone in the context of gingival recession. To our knowledge, this is the first study to evaluate alveolar trabeculation in mandibular gingival recession during orthodontic retention using FD analysis. It is expected to provide a quantitative assessment of mandibular trabecular bone changes in the retention period and clarify their relationship to gingival recession.

The primary aim of this study was to evaluate changes in alveolar trabeculation in the mandibular incisor-canine and premolar regions with and without gingival recession at the retention period following orthodontic treatment using FD analysis. The second aim of this study was to determine whether changes in FD values were correlated with the IMPA, as well as the PI and GI. The first null hypothesis of the study was that the presence or absence of gingival recession had no effect on the FD values of the alveolar trabecular structures. The second null hypothesis of the study was that there was no correlation between changes in FD values and the IMPA as well as the PI and GI.

## 2. Materials and Methods

### 2.1. Study Design, Ethics, and Consent

The study was designed as a single-center, retrospective, cross-sectional, and case–control study. Prior to the study, ethical approval was granted by Zonguldak Bülent Ecevit University Non-Interventional Clinical Research Ethics Committee on 22 January 2025 with protocol number 2025/02-11. Informed consent was obtained from all patients before the commencement of the study, and due to the retrospective nature of the study, no further consent was required.

### 2.2. Sample, Groups, and Criteria

This retrospective study included patients who presented to the Department of Periodontology at Zonguldak Bülent Ecevit University with gingival recession during the retention period following orthodontic treatment. The sample size of the study was calculated using the G*Power program (version 3.1.9.7, Franz Faul, University of Kiel, Kiel, Germany) based on the study by Aktuna Belgin and Serindere [15]. Accordingly, with an α error probability of 0.05 (α err prob) and a power of 0.95 (1-β err prob), the actual power of the study was calculated to be 0.9501803 if at least 56 samples (14 per group) were included (non-centrality parameter λ = 18.5405434; a critical F = 2.7826004).

To further increase the power of the study, one more patient was added to each group, resulting in a total of 60 samples with fifteen patients per group, divided into 4 groups: left incisor-canine region, Group 1 (10 females and 5 males, mean age 22.53 ± 2.82); right incisor-canine region, Group 2 (9 females and 6 males, mean age 22.4 ± 2.89); left premolar region, Group 3 (9 females and 6 males, mean age 21.46 ± 2.32); and right premolar region, Group 4 (7 females and 8 males, mean age 21.26 ± 2.82). The contralateral regions without gingival recession in each group were used as control regions and subsequently constituted as Group 1c, Group 2c, Group 3c, and Group 4c, respectively. Inclusion criteria for the study are as follows:

- Completed non-extraction fixed orthodontic treatment (with incisor crowding/spacing ≤ 3 mm) and in at least the first year of the retention period;
- No missing permanent teeth in the mandible except for the third molars;
- No orthodontic relapse and deformed fixed lingual retention appliance;
- No history of periodontitis;
- Presence of gingival recession ≥ 1 mm in at least one tooth (premolar or incisor-canine) in the relevant mandibular ROIs;
- No gingival recession in the contralateral region of the gingival recession site;
- No history of systemic bone disease or trauma.

High-resolution and good-quality panoramic radiographs were obtained before orthodontic treatment (T0), at the end of treatment (T1), and during the retention period (T2).

Patients who did not meet at least one of the inclusion criteria, who had undergone fixed orthodontic treatment with the extraction of permanent teeth in the mandible, and who had a history of periodontitis were excluded from the study.

### 2.3. Periodontal Examination

The periodontal examination and demographic records of patients who were referred to the periodontology clinic for gingival recession during the T2 period were retrieved from the clinical archives. Gingival recession was assessed by measuring the distance from the cemento-enamel junction to the gingival margin. Patients’ recorded plaque index (PI) and gingival index (GI) scores were accessed by scanning the clinical archives. The UNC-15 periodontal probe (CPU 15 UNC, Hu-Friedy^®^, Chicago, IL, USA) was used during these procedures, and all periodontal examinations were performed by experienced periodontists (MKÇ and RÇ). The control of the mandibular fixed retention appliances bonded to the lingual surfaces of the lower intercanine teeth was performed by an experienced orthodontist (OC), and no incisor relapse or deformation of the fixed retention appliances was observed.

Both the PI and GI were calculated separately by averaging the scores obtained from the mesiobuccal, distobuccal, midbuccal, and midlingual/palatal regions of each tooth. The definitions of PI, GI, and gingival recession are presented in Table 1 [40,41].

### 2.4. Radiograph Acquisition and Cephalometric Measurements

Panoramic radiographs of the patients were acquired separately at T0, T1, and T2 after ensuring correct positioning of the bite stick and parallel alignment of the Frankfurt horizontal plane to the floor, using an X-ray device (Veraviewepocs 2D, J Morita Mfg. Corp., Kyoto, Japan) with a range of 60–70 kVp and 1–7.5 mA. Lateral cephalometric radiographs were obtained using an X-ray device (Veraviewepocs 2D, J Morita Mfg. Corp., Kyoto, Japan) with a range of 90 kVp and 6–7 mA. The IMPAs, which represent the angle between the mandibular plane (a line passing through the Gonion and Menton points) and the long axis of the mandibular central incisor, were measured in the lateral cephalometric radiographs using the NemoCeph (updated version 2021, NemoStudio, Nemotec SL, Madrid, Spain) digital analysis program.

### 2.5. Fractal Dimension Analysis

**Figure 1:**
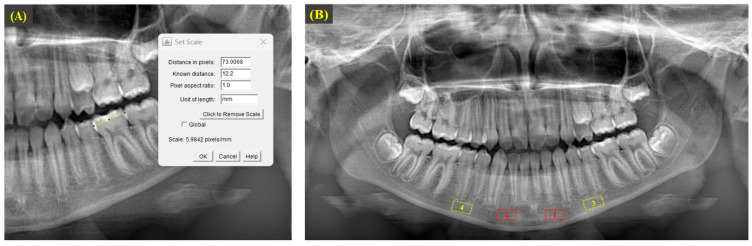
(**A**) Yellow line: calibration process for patient with mesiodistal distance of 12.2 mm for mandibular left first permanent molar. (**B**) Red-colored dashed-line mandibular ROIs are as follows: (1) left incisor-canines for Group 1; (2) right incisor-canines for Group 2. Yellow-colored dashed-line mandibular ROIs are as follows: (3) left premolars for Group 3; (4) right premolars for Group 4.

Fractal dimension analysis was performed on panoramic radiographs using ImageJ (version 1.53), a Java-based (version 8) image processing software developed by the National Institutes of Health, on the same computer (ASUS TUF Gaming F15 FX507ZC4_FX507ZC4, ASUSTeK Computer Inc., Taipei, Taiwan) by the same researcher (OC) using the box-counting method developed by White and Rudolph [42]. FD analyses were performed separately at T0, T1, and T2. To ensure methodological standardization, the mesiodistal distances of the patients’ orthodontic study casts were measured with a digital caliper (Insize digital caliper, Insize Co., Loganville, GA, USA) and, prior to FD analysis, calibration was performed in ImageJ software based on the mesiodistal distances of the teeth on panoramic radiographs of the same format. The FD analysis was performed by a researcher (OC) with at least 10 years of experience in the field and several publications on FD analysis [20,43,44]. The researcher was blinded to the regions with and without gingival recession prior to the FD analyses (see Figure 1).

**Figure 2:**
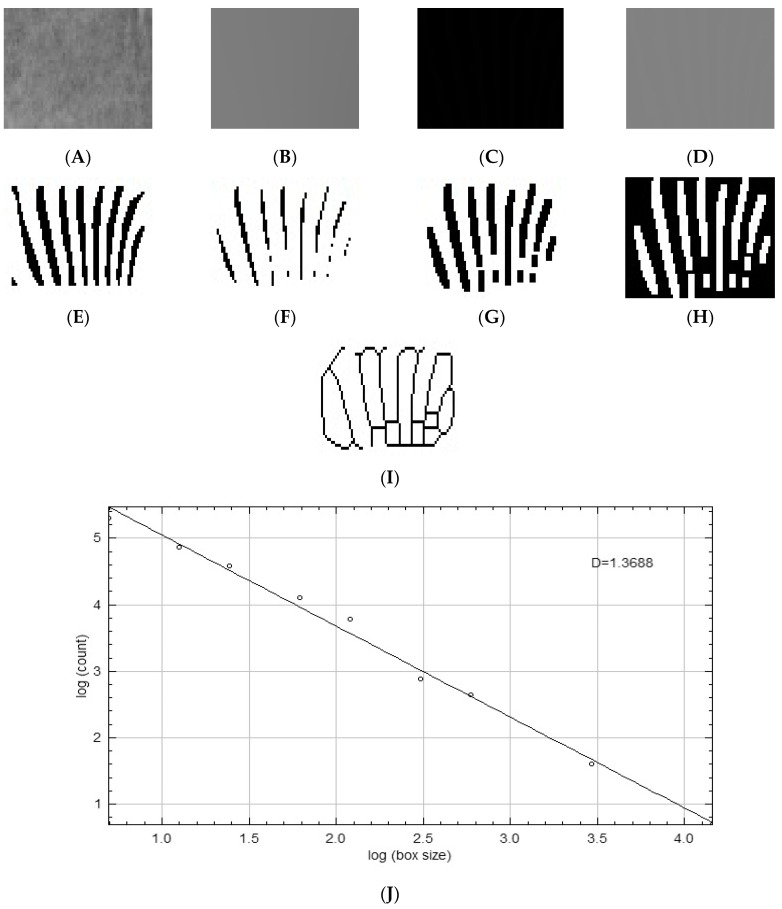
(**A**) Duplicated ROI image (70 × 35 pixels) selected from original panoramic radiograph, (**B**) image blurred with Gaussian filter, (**C**) subtraction of blurred image from original image, (**D**) image with 128 gray value added (Math), (**E**) image converted to black and white by binary (Binary), (**F**) image with minor noise removed (Erode), (**G**) image with enhanced and expanded prominent areas (Dilate), (**H**) inversion of processed image (Invert), (**I**) skeletal image obtained (Skeletonize), and (**J**) image of fractal box count (in image, D: calculated FD value for subject).

After calibration, ROIs with a size of 70 × 35 pixels (70 pixels in width and 35 pixels in height) were defined separately on the right and left sides in the apical regions of the mandibular incisor-canine and premolar teeth in all panoramic radiographs (see Figure 1). After selecting and saving the designated area in 8-bit format, a Gaussian blur filter with a sigma value of 35 pixels was applied to the copied image to eliminate factors that contribute to brightness imbalance, such as soft tissue. Through the subtraction process with the original image, the resulting image was converted to binary format by incorporating 128 gray values. To minimize the noise present in the image, a sequence of operations was performed, starting with erosion, followed by dilation, and then the application of the invert function. The skeletonize option was then applied to the image to enhance the trabecular bone structure. The final step in the process was to calculate an FD value by performing an FD analysis on the skeletal pattern image using the box-counting method [43]. These procedures were performed separately for regions with and without gingival recession (see Figure 2).

### 2.6. Statistical Analysis

Statistical analyses were performed using SPSS Statistics (version 26, IBM Corporation, Armonk, NY, USA). Normality distribution of the data was assessed using the Shapiro–Wilk test. Accordingly, time-dependent changes were assessed using one-way repeated ANOVA for parametric data or Friedman’s test for nonparametric data. Kruskal–Wallis test was used for comparisons between groups, and Chi-square test was used for categorical variables. For pairwise comparisons, *t*-test and Mann–Whitney U test were used. Pearson’s correlation was used for normally distributed data, and Spearman’s test was used for non-normally distributed data to analyze the correlation between FD, IMPA differences, PI, and GI. To assess method error, intraclass correlation coefficients (ICCs) were evaluated to determine the reliability of measurements made on 25% of the samples with a two-week interval. The level of statistical significance was set at *p* < 0.05.

## 3. Results

To assess measurement error, repeated measurements were performed on 25% of the samples with a two-week interval, demonstrating high reliability with ICC values ranging from 0.915 to 0.955 (*p* < 0.001). The data of a total of 60 patients (25 males, 35 females; mean age: 21.91 ± 2.59 years) were collected from the archive of the periodontology department. In these patients, one side had gingival recession in the mandibular incisor-canine or premolar region after a mean retention period of 1.95 ± 1.13 years following non-extraction fixed orthodontic treatment, while the contralateral side, which did not have gingival recession, was considered the control region.

No significant differences were found between the groups in terms of age (*p* = 0.545), gender distribution (*p* = 0.728), retention duration (*p* = 0.755), PI (*p* = 0.204), or GI (*p* = 0.641). The demographic data of the subjects, including age, gender, the duration of post-orthodontic retention, PI, and GI, are summarized in Table 2 by group.

**Figure 3:**
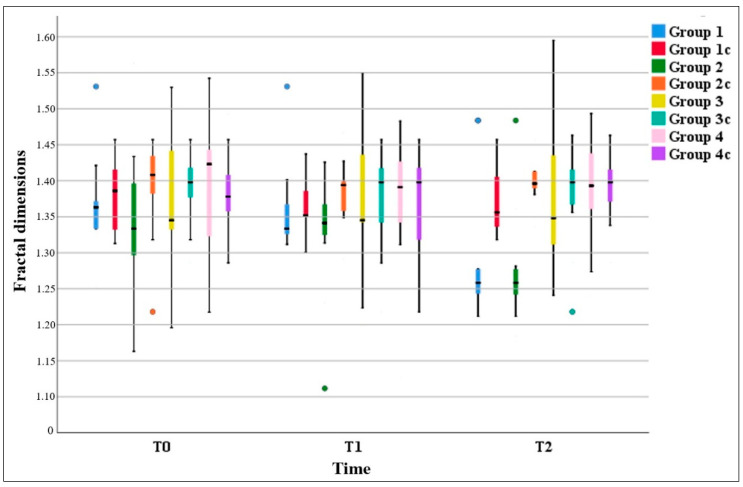
Box and whisker plot of FD value changes in groups over time.

The statistical analysis results of the within-group FD values over time for the study regions with gingival recession (Groups 1, 2, 3, and 4) and the control regions without gingival recession (Groups 1c, 2c, 3c, and 4c) are presented in Table 3. The FD values showed a statistically significant decrease over time in Group 1 (*p* = 0.001) and Group 2 (*p* = 0.003), while no significant change was observed in the control regions of Group 1c and Group 2c (*p* > 0.05). In the premolar region (Group 3, Group 3c, Group 4, and Group 4c), the FD values of both gingival recession and non-recession regions remained stable over time without significant changes (*p* > 0.05). Time-dependent pairwise comparisons within groups revealed that in Group 1, FD significantly decreased from T0 to T2 (*p* = 0.005) and from T1 to T2 (*p* = 0.011). Similarly, in Group 2, FD decreased significantly from T0 to T2 (*p* = 0.041) and from T1 to T2 (*p* = 0.027). However, no statistically significant differences were found in the time-dependent changes in FD values in Group 3, Group 3c, Group 4, and Group 4c (*p* > 0.05). The box and whisker plot of FD values at T0, T1, and T2 for regions with gingival recession (Groups 1, 2, 3, and 4) and without gingival recession (Groups 1c, 2c, 3c, and 4c) is presented in Figure 3.

At the same time points, the FD values for Group 1 and Group 2 were significantly lower than those of the controls at T2 (*p* = 0.001 for both). Notably, Group 2 also showed a significant difference compared to the controls at T1 (*p* = 0.01). In contrast, no significant differences were observed between the premolar regions (between Group 3 and Group 3c and between Group 4 and Group 4c) at any time point (*p* > 0.05) (Table 4).

The results of the pairwise statistical comparisons for the same regions in the gingival recession groups (Group 1 and Group 2; Group 3 and Group 4) are shown in Table 5. Accordingly, no significant difference was found between the pairwise comparisons of Group 1 and Group 2 at any time point (*p* > 0.05). Similarly, the differences in FD values at different time points for Group 3 and Group 4 were not statistically significant (*p* > 0.05).

The differences in FD value changes showed a significant positive correlation with both the PI (*p* = 0.037) and the GI (*p* = 0.035) (Table 6), suggesting that greater changes in FD value (decrease from baseline) were associated with higher plaque and gingival inflammation scores. However, no significant correlation was found between IMPA differences and PI, GI, or FD differences (*p* > 0.05).

## 4. Discussion

In this study, changes in alveolar trabecular bone associated with gingival recession in the mandibular anterior and premolar regions during the post-orthodontic retention period were investigated using FD analysis and compared with the contralateral regions without gingival recession. The results revealed a significant decrease in FD values over time in the anterior region (Groups 1 and 2), while FD changes in the premolar region (Groups 3 and 4) were not significant. Similarly, time-dependent FD changes in the regions without gingival recession were not found to be statistically significant. In addition, significant correlations were found between the differences in FD values (decreases) and the PI and GI scores. This finding highlights a potential relationship between alveolar bone complexity and periodontal health in regions affected by gingival recession in the retention period. The presence of gingival recession led to a decrease in FD values in the mandibular anterior region while having no effect on the premolar regions, which resulted in the rejection of the first null hypothesis in the incisor-canine region and acceptance in the premolar region. However, the first null hypothesis was accepted in the control group’s regions without gingival recession.

Fractal dimension analysis is a well-established method for detecting subtle changes in bone microarchitecture. This technique can reveal changes such as a reduction in internal trabecular structure, the reshaping of connecting trabeculae, and the thinning of the cortical bone. While these structural changes may not be clinically apparent, they can be seen on radiographic images [45]. Various approaches, including pixel dilation, mass–radius relation, and the box-counting method, are used in FD analysis [46]. In a 2020 meta-analysis, Kato identified the box-counting method as the most widely used and current approach in FD analysis [47]. In the present study, the box-counting method was chosen because it allows for comparisons with previous research and is recognized as a contemporary technique.

Previous studies suggest that FD analysis is a valuable tool for detecting early-stage periodontal disease by identifying changes in bone microstructure [15,16,48]. Şener et al. [16] reported that FD analysis is effective in quantitatively and objectively identifying early bone structural changes associated with periodontal bone loss. It appears that studies [15,16,48] reporting lower FD values in alveolar trabeculation due to periodontal disease were performed using periapical radiographs of mandibular first molars [15,16,48]. In contrast, most studies showing no change in FD with periodontal status were based on panoramic radiographs, and it has been reported that these inconsistencies in the literature may be due to differences in imaging techniques [49,50]. In our study, panoramic measurements showed high reliability in repeated assessments, with high ICC values ranging from 0.915 to 0.955.

There are studies that report negative correlations between FD and various periodontal clinical parameters, such as clinical attachment level, probing depth, and bleeding on probing percentage [48,51]. On the other hand, there are studies that found no significant relationship between gender and FD [15,48,52], similarly, Amer et al. [53] showed that the FD of trabecular bone was not associated with age. In our study, because of the homogeneous distribution of age and gender, the observed FD changes were not due to demographic differences, and decreasing FD values were observed with increasing PI and GI values.

In a retrospective case–control study of a sample with a 10-year retention period following orthodontic treatment, no significant changes in FD were observed in mandibular anterior incisors from pretreatment to the end of retention in either the relapse or stable treatment groups [54]. In contrast, another study reported FD changes particularly distal to the second premolar and anterior to the mental foramen following functional orthopedic treatment [28]. Cicek and Arslan [20] reported that functional treatment did not cause a significant change in FD of the symphysis, but the significant decrease in FD observed in the alveolar bone buccal to the mandibular incisors was due to the mandibular incisor protrusion induced by functional treatment.

Mandibular incisor crowding can be resolved by interproximal enamel reduction on the incisors or by their protrusion through orthodontic tooth movement [55]. Excessive proclination of mandibular incisors has been reported to result in poor esthetics, relapse, and gingival recession [55]. During the initial phase of fixed orthodontic treatment, protrusive forces from the bracket prescription, especially from the mesial tip of the canines, induce proclination of the mandibular incisors [55,56]. However, Hennessy et al. [55] reported that mild mandibular incisor protrusion occurs with fixed orthodontic treatment in cases of mandibular incisor crowding. In addition, other studies in the literature report that labial movement of mandibular incisors is not a risk factor for gingival recession [57,58]. Heterogeneity in study results may be due to differences in orthodontic treatment protocols between populations, as well as the selection of different ROIs, which limit the generalizability of orthodontic treatment-related changes in FD [20,21]. Our results show a significant correlation between FD and both PI and GI scores, while no correlation was found with IMPA, indicating that the significant decrease in FD in anterior recession regions is associated with higher PI and GI values. This situation led to the acceptance of the second null hypothesis between FD and the IMPA, while it was rejected between FD and the PI and GI.

Pandis et al. [59] reported that there is no clear evidence of a direct relationship between the presence of fixed retention appliances and the occurrence of gingival recession. However, it has been noted that long-term fixed retention appliances, particularly in the presence of protruded mandibular incisors, may result in loss of attachment [36,59]. The literature on the prevalence of gingival recession in orthodontically treated and untreated patients is conflicting [8,33,60], with Thomson [60] reporting no effect of orthodontic treatment on gingival recession, whereas Slutzkey and Levin [8], along with Renkema et al. [33], reported a higher prevalence of gingival recession in orthodontically treated patients compared with untreated controls. It has been proposed that these conflicting results may be due to differences in periods of observation or differences in the characteristics of the control groups in these studies [36]. The mean retention period in our study was 1.95 ± 1.13 years, with no retention appliance deformation or incisor relapse. This was to minimize gingival recession due to early post-treatment complications or long-term periodontal deposits. Thus, our results on FD values and orthodontic–periodontal parameters were based on an optimally standardized sample.

According to a recent study, the prevalence of gingival recession in the mandibular anterior region during the retention period following orthodontic treatment is 38% [31]. These gingival recessions can occur due to various etiological factors, such as periodontal phenotype, periodontal disease, age, and orthodontic treatment [61]. Although the identification of these factors is critical to the diagnosis, treatment, and prevention of gingival recession, our current understanding of the structural changes in hard and soft tissues following gingival recession remains limited. Most studies evaluating the influence of hard tissues on gingival recession have focused primarily on alveolar bone thickness without adequately examining features of bone microarchitecture such as the trabecular structure and density [45]. Our results show a significant decrease in FD in the anterior mandible with gingival recession, indicating trabecular loss, whereas FD changes in the premolar region were minimal and not significant. This suggests that the thicker cortical bone and favorable anatomy of the premolar region may provide greater resistance to structural changes.

Studies investigating the effect of periodontal pathology on FD values have reported variable results due to differences in ROIs, sizes, and imaging techniques [15,16,48]. Methodological standardization and careful ROI selection are consistently emphasized in FD analysis research [15,16,48]. Our study is the first case–control study to evaluate FD values in gingival recession in the post-orthodontic retention period. The split-mouth design, including both recession and control regions, increases reliability by minimizing individual variability. However, the lack of CBCT-based analysis, which could provide more detailed insights into FD changes, and the focus on patients with fixed lingual retainers are only limitations of our study. Further research is needed to include cases treated with removable retention appliances. Nevertheless, our findings highlight the need for orthodontists and periodontists to work together to evaluate patients with compromised periodontal prognosis, particularly in the anterior mandible.

## 5. Conclusions

Within the limitations of the study, it was concluded that (i) FD analysis can effectively assess alveolar trabeculation changes in gingival recessions, particularly in the mandibular anterior region; (ii) FD values decrease with declining oral hygiene motivation and increasing PI and GI scores; (iii) changes in the IMPA show no correlation with FD value alterations; and (iv) the frequency of periodontal check-ups should be increased post-orthodontic treatment to ensure long-term periodontal health.

## Figures and Tables

**Table 1:** Definitions of PI, GI, and gingival recession.

Parameter	Score	Definition
For GI	0	Healthy gingiva
	1	Mild infection with color change and edema without bleeding on probing
	2	Moderate infection with color change and edema, along with bleeding on probing
	3	Severe infection with marked edema and hyperemic gingiva with tendency to spontaneously bleed
For PI	0	No plaque
	1	Presence of film-like plaque, not visible by direct observation but detectable by periodontal probing, located at free gingival margin and on relevant tooth surface
	2	Presence of visible soft deposits at gingival margin and on tooth surface
	3	Presence of excessive soft deposits at gingival margin and on tooth surface
For gingival recession		Distance measured from cemento-enamel junction to gingival margin on exposed root surface that exceeds 0.5 mm

GI: gingival index; PI: plaque index.

**Table 2:** The data of the patients included in the study.

		Group 1	Group 2	Group 3	Group 4	*p*
Age (year)	Mean ±SD	22.53 ± 2.82	22.4 ± 2.89	21.46 ± 2.32	21.26 ± 2.28	0.545 ^K^
Gender	Female, *n* (%)	10 (67)	9 (60)	9 (60)	7 (47)	0.728 ^χ2^
	Male, *n* (%)	5 (33)	6 (40)	6 (40)	8 (53)	
Retention duration (year)	Mean ± SD	1.98 ± 1.36	1.88 ± 1.25	1.91 ± 0.9	2.06 ± 1.07	0.755 ^K^
PI	Mean ± SD	1.93 ± 0.79	1.93 ± 0.88	2.46 ± 0.99	2.2 ± 0.94	0.204 ^K^
GI	Mean ± SD	1.33 ± 0.81	1.26 ± 0.7	1.46 ± 0.51	1.4 ± 0.5	0.641 ^K^

SD: standard deviation, PI: plaque index, GI: gingival index. ^K^: Kruskal–Wallis test, ^χ2^: Chi-square test, *p*: significance level.

**Table 3:** Statistical analysis results of time-dependent FD changes in groups.

	T0	T1	T2	*p*	T1-T0	T2-T1	T2-T0
	Mean ± SD (Median)	Mean ± SD (Median)	Mean ± SD (Median)		*p*	*p*	*p*
Group 1	1.3680 ± 0.059 (1.3631)	1.3517 ± 0.060 (1.3336)	1.2834 ± 0.084 (1.2583)	0.001 *^,F^	0.079 ^W^	0.011 *^,W^	0.005 *^,W^
Group 1c	1.3812 ± 0.052 (1.386)	1.3626 ± 0.041 (1.352)	1.3752 ± 0.47 (1.356)	0.262 ^A^	0.15 ^A^	1.000 ^A^	0.852 ^A^
Group 2	1.3546 ± 0.11 (1.3336)	1.3303 ± 0.075 (1.3412)	1.2703 ± 0.073 (1.2583)	0.003 *^,F^	0.326 ^W^	0.027 *^,W^	0.041 *^,W^
Group 2c	1.3959 ± 0.062 (1.408)	1.3836 ± 0.039 (1.394)	1.4013 ± 0.035 (1.396)	0.119 ^F^	0.147 ^W^	0.125 ^W^	0.878 ^W^
Group 3	1.3792 ± 0.104 (1.3452)	1.3760 ± 0.095 (1.3412)	1.3787 ± 0.105 (1.3482)	0.991 ^A^	0.874 ^A^	0.918 ^A^	1.000 ^A^
Group 3c	1.3919 ± 0.051 (1.398)	1.3772 ± 0.065 (1.396)	1.3852 ± 0.064 (1.397)	0.220 ^A^	0.211 ^A^	1.000 ^A^	0.786 ^A^
Group 4	1.3809 ± 0.098 (1.4232)	1.3789 ± 0.87 (1.3912)	1.3841 ± 0.67 (1.3932)	0.627 ^F^	0.733 ^W^	0.496 ^W^	0.955 ^W^
Group 4c	1.3819 ± 0.054 (1.378)	1.3732 ± 0.07 (1.398)	1.3892 ± 0.054 (1.388)	0.413 ^A^	0.568 ^A^	1.000 ^A^	0.097 ^A^

SD: standard deviation, T0: before treatment, T1: end of treatment, T2: retention period, ^A^: one-way repeated measures ANOVA test, ^F^: Friedman test, ^w^: Wilcoxon signed-rank test, *p*: significance level, *: *p* < 0.05.

**Table 4:** Pairwise comparison results of groups with controls at same time points.

	T0	*p*	T1	*p*	T2	*p*
	Mean ± SD (Median)		Mean ± SD (Median)		Mean ± SD (Median)	
Group 1	1.3680 ± 0.059 (1.3631)	0.524 ^t^	1.3517 ± 0.060 (1.3336)	0.285 ^M^	1.2834 ± 0.084 (1.2583)	0.001 *^,t^
Group 1c	1.3812 ± 0.052 (1.386)		1.3626 ± 0.041 (1.352)		1.3752 ± 0.047 (1.356)	
Group 2	1.3546 ± 0.11 (1.3336)	0.218 ^t^	1.3303 ± 0.075 (1.3412)	0.01 *^,M^	1.2703 ± 0.073 (1.2583)	0.001 *^,t^
Group 2c	1.3959 ± 0.062 (1.408)		1.3836 ± 0.039 (1.394)		1.4013 ± 0.035 (1.396)	
Group 3	1.3792 ± 0.104 (1.3452)	0.676 ^t^	1.3760 ± 0.095 (1.3412)	0.969 ^t^	1.3787 ± 0.105 (1.3482)	0.841 ^t^
Group 3c	1.3919 ± 0.051 (1.398)		1.3772 ± 0.065 (1.396)		1.3852 ± 0.064 (1.397)	
Group 4	1.3809 ± 0.098 (1.4232)	0.973 ^t^	1.3789 ± 0.87 (1.3912)	0.539 ^M^	1.3841 ± 0.67 (1.3932)	0.821 ^t^
Group 4c	1.3819 ± 0.054 (1.378)		1.3732 ± 0.07 (1.398)		1.3892 ± 0.054 (1.388)	

SD: standard deviation, T0: before treatment, T1: end of treatment T2: retention period. ^M^: Mann–Whitney U test, ^t^: *t* test, *p*: significance level, *: *p* < 0.05.

**Table 5:** Inter-group comparison results at same time points for same regions with gingival recession.

	T0	*p*	T1	*p*	T2	*p*
	Mean ± SD (Median)		Mean ± SD (Median)		Mean ± SD (Median)	
Group 1	1.3680 ± 0.059 (1.3631)	0.449 ^M^	1.3517 ± 0.060 (1.3336)	0.983 ^M^	1.2834 ± 0.084 (1.2583)	0.786 ^M^
Group 2	1.3546 ± 0.11 (1.3336)		1.3303 ± 0.075 (1.3412)		1.2703 ± 0.073 (1.2583)	
Group 3	1.3792 ± 0.104 (1.3452)	0.964 ^t^	1.3760 ± 0.095 (1.3412)	0.933 ^t^	1.3787 ± 0.105 (1.3482)	0.869 ^t^
Group 4	1.3809 ± 0.098 (1.4232)		1.3789 ± 0.87 (1.3912)		1.3841 ± 0.67 (1.3932)	

SD: standard deviation, T0: before treatment, T1: end of treatment, T2: retention period. ^M^: Mann–Whitney U test, ^t^: *t* test, *p*: significance level.

**Table 6:** Correlation analysis results for FD value and IMPA differences.

		FD Differences	PI	GI
FD differences	Mean ± SD	−0.002 ± 0.12	2.13 ± 0.91	1.43 ± 0.69
	Pearson coefficient	1	0.269	0.273
	*p*		0.037 *	0.035 *
IMPA differences	Mean ± SD	−0.06 ± 0.12	1.93 ± 0.82	1.3 ± 0.74
	Spearman rho	−0.106	0.161	0.148
	*p*	0.578	0.397	0.436

SD: standard deviation, PI: Plaque index, GI: Gingival index, IMPA: Incisor mandibular plane angle *p*: significance level, *: *p* < 0.05.

## Data Availability

The datasets used in this study are accessible from the corresponding author upon reasonable request.
